# A Novel Nomogram for Predicting the Risk of Short-Term Recurrence After Surgery in Glioma Patients

**DOI:** 10.3389/fonc.2021.740413

**Published:** 2021-10-26

**Authors:** Tianwei Wang, Chihao Zhu, Shuyu Zheng, Zhijun Liao, Binghong Chen, Keman Liao, Xi Yang, Zhiyi Zhou, Yongrui Bai, Zhenwei Wang, Yanli Hou, Yongming Qiu, Renhua Huang

**Affiliations:** ^1^Department of Neurosurgery, Renji Hospital, Shanghai Jiao Tong University School of Medicine, Shanghai, China; ^2^Department of Oncology Radiation, Shanghai International Medical Center, Shanghai, China; ^3^Department of Radiation, Renji Hospital, Shanghai Jiao Tong University School of Medicine, Shanghai, China

**Keywords:** recurrent glioma, nomogram, short-term recurrence, extent of resection, IDH

## Abstract

**Objective:**

The aim of this study was to establish a nomogram model for predicting the risk of short-term recurrence in glioma patients.

**Methods:**

The clinical data of recurrent glioma patients were summarized and analyzed in this study. Univariate and multivariate logistic regression analyses were performed to analyze the correlation between clinical data and the risk of short-term recurrence after operation. A nomogram was established based on the multivariate logistic regression model results.

**Results:**

A total of 175 patients with recurrent glioma were enrolled, with 53 patients in the short-term recurrence (STR) group (recurrent time ≤6 months) and 122 patients in the long-term recurrence (LTR) group (recurrent time ≥36 months). Univariate analysis revealed that age at diagnosis, Karnofsky performance scores (KPSs), tumor location, glioma grade, glioma type, extent of resection (EOR), adjuvant chemotherapy (ad-CT), concurrent chemotherapy (co-CT), and isocitrate dehydrogenase (IDH) status were significantly associated with the short-term glioma recurrence. Multivariate analyses revealed that age at diagnosis, KPS, glioma grade, EOR, and IDH were independent risk factors for short-term glioma recurrence. A risk nomogram for the short-term recurrence of glioma was established, with the concordance index (C-index) of 0.971. The findings of calibration and receiver operating characteristic (ROC) curves showed that our nomogram model had good performance and discrimination to estimate short-term recurrence probability.

**Conclusion:**

This nomogram model provides reliable information about the risk of short-term glioma recurrence for oncologists and neurosurgeons. This model can predict the short-term recurrence probability and give assistance to decide the interval of follow-up or formulate individualized treatment strategies based on the predicted results. A free online prediction risk tool for this nomogram is provided: https://rj2021.shinyapps.io/Nomogram_ recurrence-risk/.

## Introduction

Glioma is the most common primary intracranial tumor, with a high mortality rate and poor outcome (1–3). Combined treatment regimens can prolong the survival of patients; however, the prognosis is still dismal, and most of tumor will recur in a few years (4–7). At present, most of studies focus on the overall survival (OS) of glioma, while there are few studies on the risk factors of tumor recurrence. As previously reported, tumor grade, treatment regimens, and the status of isocitrate dehydrogenase (IDH) are associated with survival (8–10). What is more, time to recurrence also is an independent predictor for survival (11, 12), with short-term recurrence predicting a worse prognosis; therefore, a tool that can predict short-term recurrence risk of glioma is particularly important.

In previous studies, some scholars have established several survival nomogram models for primary glioma, such as glioblastoma (GBM), lower-grade glioma, and thalamic glioma survival models; and these nomograms showed good performance and discrimination to estimate survival probability (13–18). However, no nomogram model for predicting the risk of short-term recurrence in glioma patients has been reported. A risk nomogram for predicting short-term recurrence of glioma is needed urgently, and it is a tool for clinicians to use for predicting the probability of short-term recurrence, developing an individualized management strategy after tumor recurrence, and formulating the interval of follow-up. The aim of this study is to establish and then independently validate a nomogram model for estimating individualized short-term recurrence probabilities for glioma patients, which would be readily accessible for clinical use.

## Materials and Methods

All data used in our study came from June 2008 to August 2020 in Renji Hospital, Shanghai Jiaotong University School of Medicine. The patients with recurrence time ≤6 months were defined as the short-term recurrence group, and the long-term recurrence (LTR) group was defined as recurrent time ≥36 months. A total of 175 patients diagnosed as having glioma through histopathologic analysis were included. For every patient, the following variables were obtained: age at diagnosis, sex (male or female), preoperative Karnofsky performance score (KPS), extent of resection (EOR; gross total resection (GTR), STR^a^ (subtotal resection), or partial resection (PR)), radiotherapy (RT), concurrent chemotherapy (co-CT), adjuvant chemotherapy (ad-CT), glioma grade (lower grade or high grade), glioma type (oligodendroglioma or astrocytoma), IDH status (mutation, wild type, or not otherwise specified (NOS)), *O*^6^-methylguanine-DNA methyltransferase (MGMT) promoter (methylation or unmethylation), and recurrent time (≤6 or ≥36 months) in months. This nomogram was established by the significant risk factors screened by statistics, and then we used the concordance index (C-index), calibration plots, and receiver operating characteristic (ROC) curve to validate this model. This study was approved by the ethics committees of Renji Hospital, Shanghai Jiaotong University School of Medicine, according to principles of the Declaration of Helsinki.

### Statistical Analysis

Univariate and multivariate logistic regression analyses were performed using IBM SPSS Version 23.0 software (IBM Corporation, Armonk, New York, USA). Descriptive statistics were used to characterize the population. Univariate and multivariate logistic regression analyses were used to assess the association of short-term glioma recurrence with clinical characteristics and management data, including age, sex, KPS, glioma grade, glioma type, tumor location, EOR, co-CT, ad-CT, IDH1, and MGMT promoter. The nomogram, calibration, and ROC curves were drawn with R statistical software (version 4.0.4). The model was established based on multivariable logistic regression results, and calibration curve was used to evaluate the consistency between the actual and predicted probabilities of short-term recurrence. The distinction of the nomogram for predicting short-term recurrence risk was appraised by the C-index, and the sensitivity and specificity were tested by ROC curve.

## Results

A total of 175 adult patients were enrolled (102 male and 73 female), including 118 cases of lower-grade gliomas: 53 diffuse astrocytomas (DA) 30 oligodendrogliomas (O), 17 anaplastic astrocytomas (AA), and 18 anaplastic oligodendrogliomas (AO) and 57 cases of high-grade gliomas: 57 GBM. The median age at diagnosis was 48 (range: 18–74 years), and the median KPS before surgery was 90.0 (range: 60.0–100.0). More than half of tumors (98 cases) were located at the frontal lobe. Surgery consisted of GTR/STR^a^ in 96 patients (54.9%) and PR in 70 patients (45.1%). Postoperatively, all the populations received RT, 80 patients (45.7%) received co-CT, and 95 patients (54.3%) received ad-CT.

The recurrent time of 53 patients was ≤6 months, and 122 patients had recurrent time ≥36 months. Comparing the characteristics and management data between these two groups, we found that the age at diagnosis of the STR group was lower in the LTR group (56.19 ± 13.36 *vs.* 42.43 ± 13.91, *p* < 0.001). The STR group had a lower KPS than the LTR group (81.51 ± 11.33 *vs.* 89.45 ± 5.93, *p* < 0.001). Frontal gliomas had a lower short-term recurrence rate, compared with other location’s glioma (*p* = 0.028). In addition, the glioma grade was closely associated with short-term recurrence, with high-grade glioma showing a higher short-term recurrent rate lower-grade glioma (*p* < 0.001), and the short-term recurrent rate of astrocytoma and oligodendroglioma has marked difference, with astrocytoma patients having a higher short-term recurrent rate (*p* = 0.006). And 84.9% (45/53) of patients in the STR group received PR, which was higher than in the LTR group (27.9%, *p* < 0.001; **Table 1**). Compared with STR glioma patients, more LTR glioma patients received combined therapy of RT and co-CT (31/53 *vs.* 49/122, *p* = 0.027), and there was a significant difference in RT and ad-CT between the STR and LTR groups (36/53 *vs.* 59/122, *p* = 0.018). A significant difference in status of IDH was observed between the STR and LTR groups, with 13.3% of STR (4/30) and 73.7% of LTR (14/17) showing IDH mutation (*p* < 0.001, **Table 1**).

**Table 1:** Patient demographics.

Variable		STR (n)	LTR (n)	OR	95% CI	*p*
		TTR ≤ 6 months	TTR ≥ 12 months			
Age (years)		42.43 ± 13.91	42.43 ± 13.91	0.920	0.891, 0.950	<0.001
KPS		81.51 ± 11.33	89.45 ± 5.93	1.118	1.067, 1.172	<0.001
Gender	Male	28	74	0.726	0.379, 1.392	0.335
	Female	25	48			
Location	Frontal lobe	23	75	2.081	1.082, 4.004	0.028
	Others	30	47			
Glioma type	A	52	92	16.957	2.247, 127.974	0.006
	O	1	30			
Tumor grade	Lower grade	9	109	40.991	16.349, 102.774	<0.001
	High grade	44	13			
EOR	GTR/STR^a^	8	88	0.069	0.029, 0.161	<0.001
	PR	45	34			
RT + co-CT	No	22	73	0.476	0.247, 0.917	0.027
	Yes	31	49			
RT + ad-CT	No	17	63	0.442	0.225, 0.871	0.018
	Yes	36	59			
IDH1	Mutation	4	14	0.043	0.015, 0.124	<0.001
	Wild type	26	5			
	NOS	23	103			
MGMTp	No	12	6			0.444
methylation	Yes	5	5			

STR, short-term recurrence; LTR, long-term recurrence; TTR, time to recurrence; KPS, Karnofsky performance score; EOR, extent of resection; GTR, gross total resection; STR^a^, subtotal resection; PR, partial resection; RT, radiotherapy; co-CT, concurrent chemotherapy; ad-CT, adjuvant chemotherapy; IDH1, isocitrate dehydrogenase; MGMTp, O^6^-methylguanine-DNA methyltransferase promoter; NOS, not otherwise specified.

**Figure 1:**
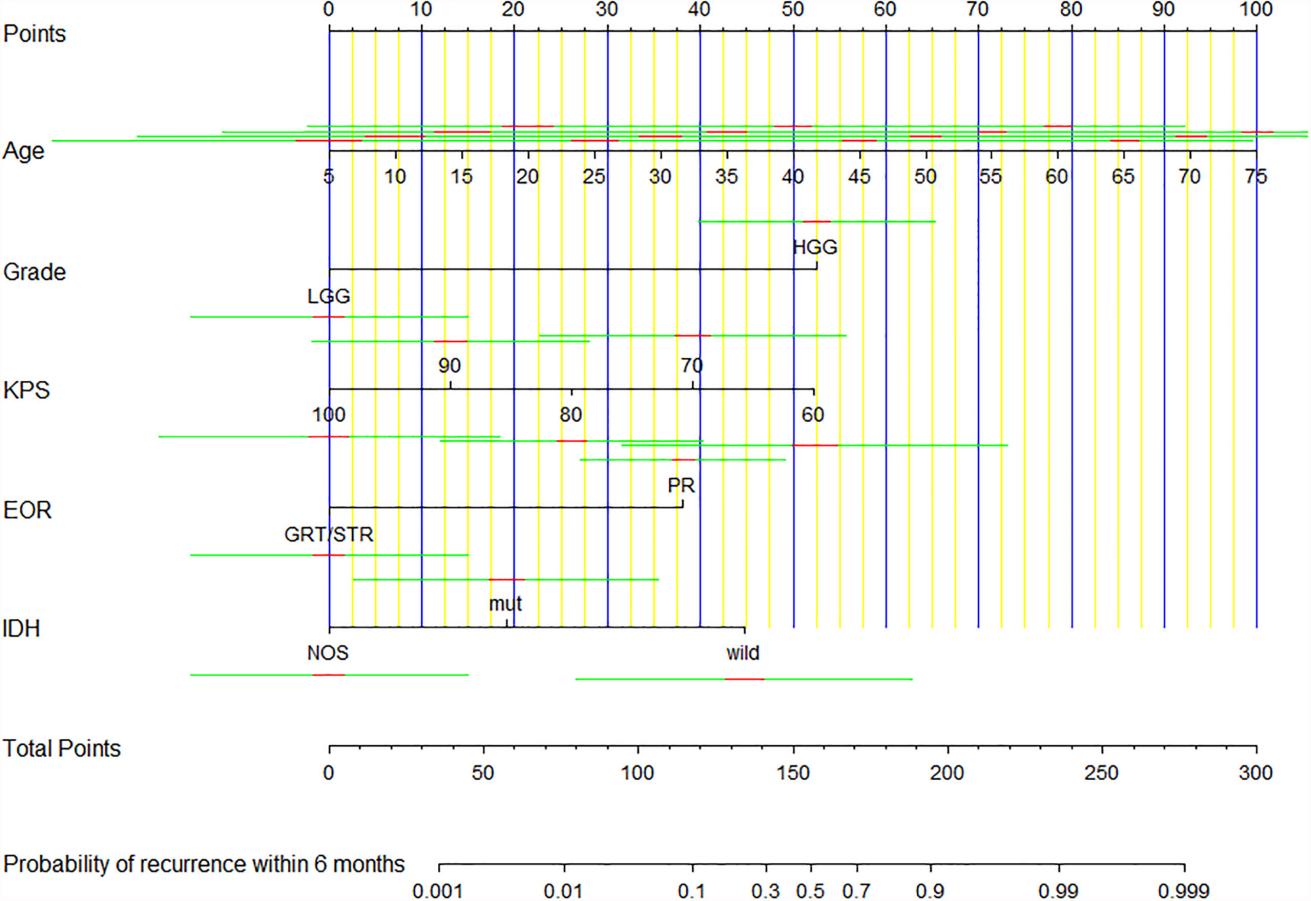
A nomogram for predicting the risk of short-term recurrence of glioma.

**Figure 2:**
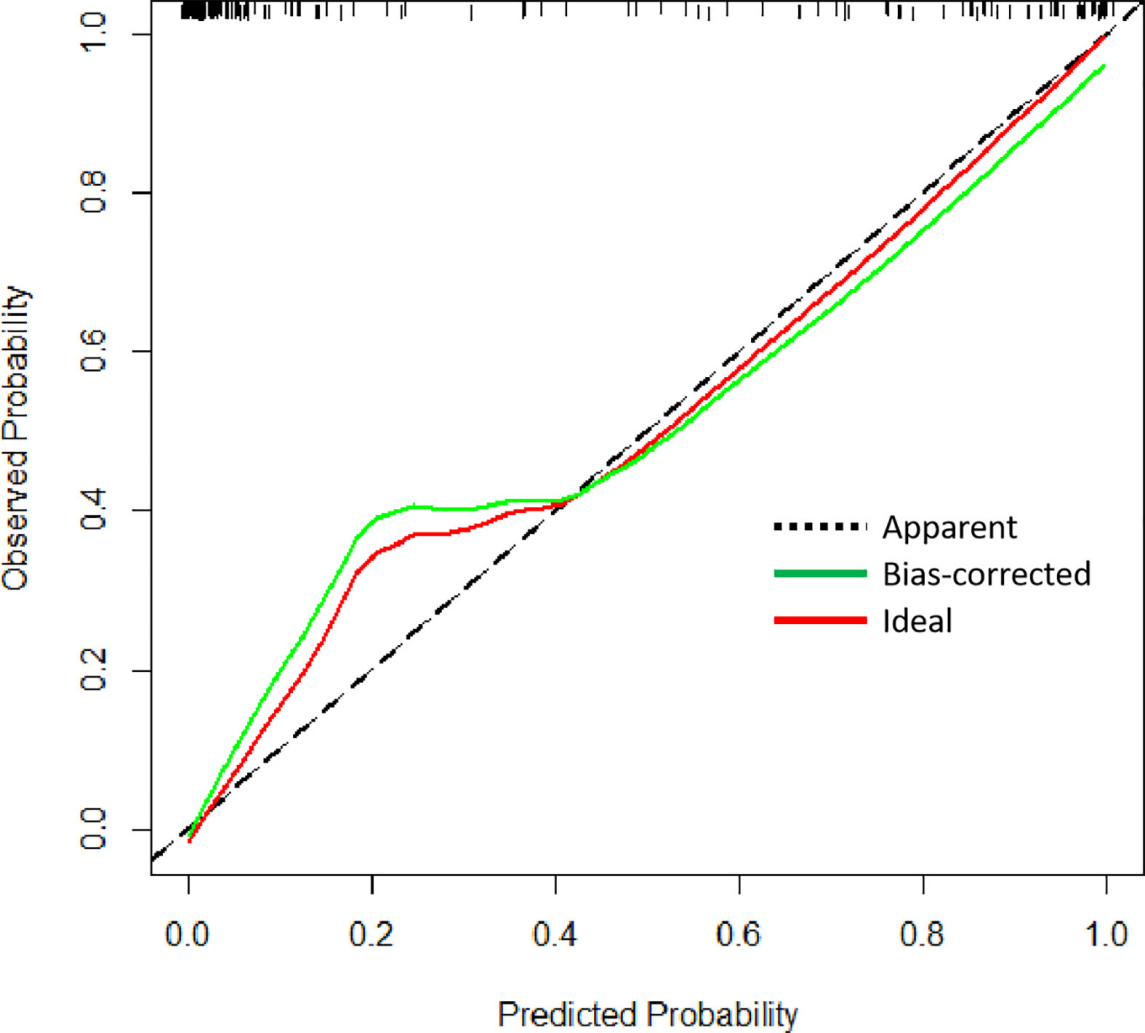
Calibration curve for the risk of short-term recurrence.

**Figure 3:**
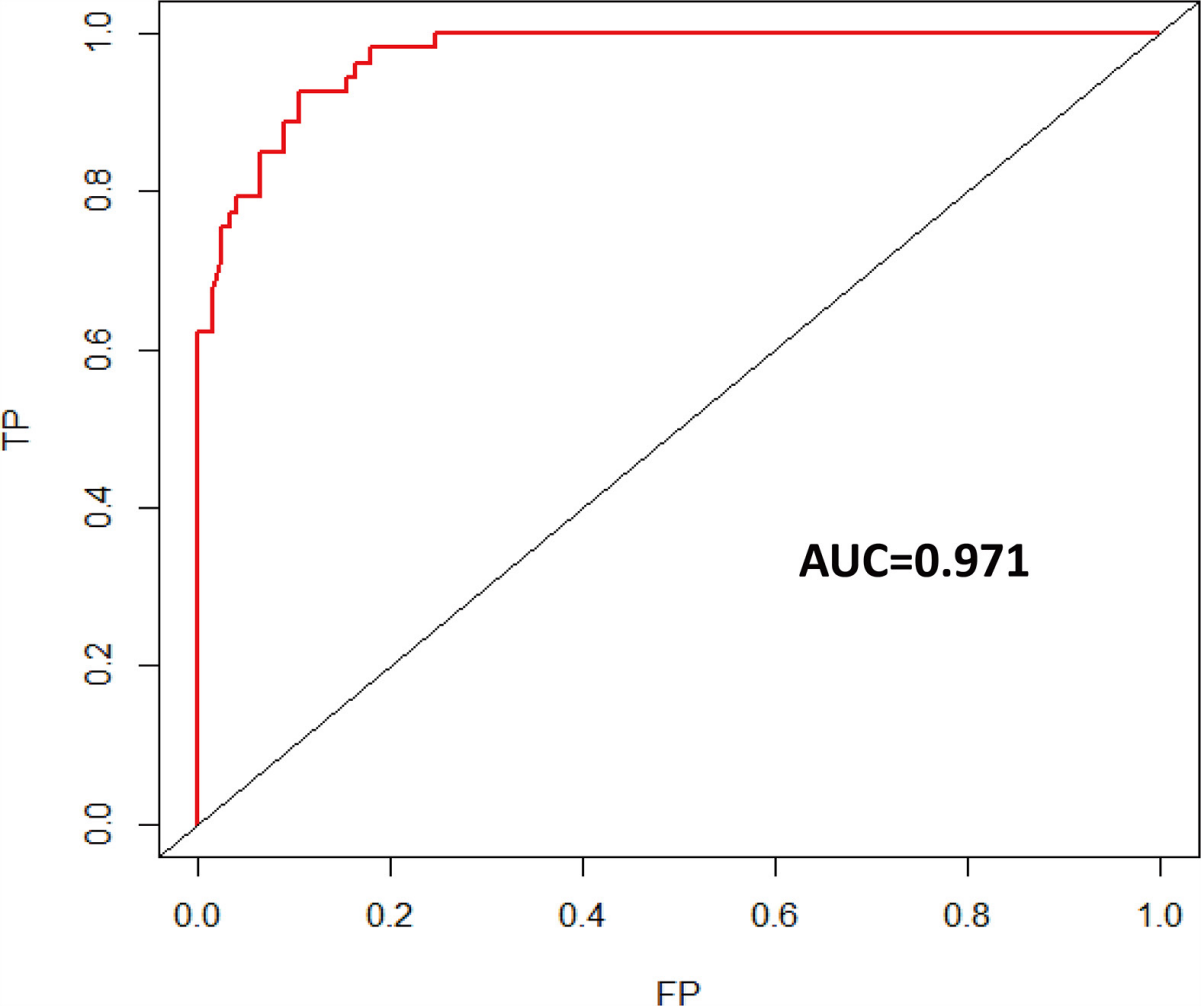
Time-dependent ROC curve of short-term recurrence (AUC = 0.971). ROC, receiver operating characteristic; AUC, area under the ROC curve.

Univariate logistic regression analysis showed that age at diagnosis, KPS, tumor grade, glioma type, tumor location, EOR, co-CT, ad-CT, and IDH1 were significantly associated with short-term glioma recurrence. However, multivariate logistic regression analysis revealed that age at diagnosis (OR 0.925, [95% CI], 0.875–0.978, *p* = 0.006), KPS (OR 1.106, [95% CI 1.023–1.196], *p* = 0.011), tumor grade (OR 17.429, [95% CI 4.618–67.790], *p* < 0.001), EOR (OR 9.894, [95% CI 2.332–41.979], *p* = 0.002), and IDH1 (OR 0.049, [95% CI 0.006–0.432], *p* = 0.007) were independent risk factors of short-term glioma recurrence (**Table 2**). A nomogram was established to predict the short-term glioma recurrent risk of glioma according to the multivariate logistic regression analysis findings (**Figure 1**). The variables that increased the probability of short-term glioma recurrent risk included older age at diagnosis, lower preoperative KPS, high-grade tumor, PR, and IDH1 wild type. The C-index of this nomogram was 0.971 (95% CI, 0.951–0.990), and there was a good agreement between the bias-corrected curve and the ideal curve in the calibration curve (**Figure 2**). What is more, the value of area under the ROC curve (AUC) was 0.971 (95% CI: 0.951–0.990, **Figure 3**), and this result showed that this nomogram had good sensitivity and specificity. These validation findings demonstrated that this risk nomogram model had a reliable predictive performance and discrimination for estimating short-term recurrence probability.

**Table 2:** Multivariate analysis for statistically significant factors.

Variable	OR	95% CI	*p*
Age (continuous)	0.925	0.875, 0.978	0.006
KPS (continuous)	1.106	1.023, 1.196	0.011
Location (frontal lobe *vs.* other locations)	1.709	0.415, 7.035	0.458
Tumor grade (lower grade *vs.* high grade)	17.429	4.618, 67.790	<0.001
Glioma type (O *vs.* A)	0.100	0.002, 5.815	0.266
EOR (GTR/STR^a^ vs. PR)	9.894	2.332, 41.979	0.002
RT + co-CT (yes *vs.* no)	0.440	0.077, 2.506	0.355
RT + ad-CT (yes *vs.* no)	3.253	0.669, 15.821	0.144
IDH1 (mutation *vs.* wild-type *vs.* NOS)	0.049	0.006, 0.432	0.007

KPS, Karnofsky performance score; EOR, extent of resection; GTR, gross total resection; STR^a^, subtotal resection; PR, partial resection; RT, radiotherapy; co-CT, concurrent chemotherapy; ad-CT, adjuvant chemotherapy; IDH1, isocitrate dehydrogenase; NOS, not otherwise specified.

## Discussion

The purpose of this study was to establish as well as validate, both internally and on an independent data, an individual short-term recurrence risk nomogram model for recurrent glioma patients. The logistic regression model was the best calibrated and fitting model to evaluate short-term recurrence rate based on a 10-fold validation C-index; these short-term recurrence risk factors included age at diagnosis, KPS, EOR, tumor grade, and IDH status. This model was validated by three methods, showing a reliable good predictive performance.

Age was considered as an independent prognostic factor for progression-free survival (PFS) and OS (9, 19–21). In our study, the short-term recurrence rate was distinct different between those two groups, with the patients in the STR group having older age. No studies have described the relationship between age at diagnosis and short-term tumor recurrence before; however, in Burgenske’s study (22), they made a comparison in survival outcome between 37 short-term survivors (OS ≤ 6 months) and 12 long-term survivors (OS ≥ 60 months), and they found that long-term survivors were younger (median age: 50 *vs.* 61 year), and their result was indirectly consistent with our findings. We concluded that older age at diagnosis was an independent risk factor for short-term recurrence.

KPS was associated with prognosis, with the patients with high KPS having prolonged survival, and it was confirmed as a survival predictor in previous papers (9, 23, 24). We found there was significant discrepancy in preoperative KPS between the STR group and LTR group, in which the STR group had a lower preoperative KPS than the LTR group (median value: 80 *vs.* 90). This result was in accordance with previous studies, indicating that low preoperative KPS was an independent risk factor for short-term recurrence of glioma.

There was no doubt that tumor grade was an independent prognostic factor for PFS, and the patients with lower-grade glioma had prolonged PFS (10). In our study, 83.0% of patients were diagnosed as having high-grade glioma in the STR group, which is much higher than that in the LTR group (10.7%); this finding was consistent with relative published papers (9). Our analysis also showed that glioma type and location had a relationship with short-term recurrence, showing that patients with oligodendroglioma and frontal lobe tumor have lower short-term recurrence rate as compared with astrocytoma and other locations glioma, respectively; however, they were not independent risk factors for short-term recurrence by multivariate logistic regression analysis.

The maximal resection that was safely feasible was the preferred guiding principle for any type of gliomas, and EOR was closely associated with prognosis (8, 24–27). However, it was difficult to achieve complete resection for some glioma patients due to the invasive growth of tumor, which could lead to glioma recurrence at a short-term time. Gross total or subtotal resection was performed in only 15.1% of patients in the STR group; however, 72.1% of patients in the LTR group received gross total or subtotal resection; this result indirectly indicated that there was a close relationship between EOR and short-term recurrence as a related previous paper has reported (10). In Rossi’s study, they found that almost all patients with PR suffered from tumor recurrence, to some extent (10); their result was similar with our finding that the patients with PR in the STR group had a high short-term recurrence risk, and PR was confirmed as an independent risk factor for short-term recurrence. In addition, we also found that co-CT and ad-CT were associated with short-term recurrence; however, these two factors were not independent risk factors.

IDH1 was a key rate-limiting enzyme in the Krebs cycle (28, 29), and IDH1 mutation was a favorable independent prognostic factor for PFS in glioma (9, 10), and it also played an important role in the time of tumor recurrence and outcome of gliomas. In previous studies, the patients with IDH1 mutation had prolonged PFS (30–32). Our study showed the IDH1 mutation rate in the STR group was 13.3%, which was extremely lower than in the LTR group (73.7%), and IDH1 wild type was confirmed as an independent risk factor for short-term glioma recurrence. Although no studies have explicitly compared the difference in IDH1 status between STR and LTR patients, Weller et al. (9) made a survival analysis for 286 patients with newly diagnosed GBM; they found that IDH1 mutation had a relationship with prolonged PFS; their result was indirectly consistent with our finding, suggesting that IDH1 wild type was an independent short-term recurrence risk factor for glioma patients to some extent.

Age at diagnosis, KPS, tumor grade, EOR, and IDH status had a close relationship with short-term recurrence, and these variables were independent risk factors for short-term recurrence in glioma. Based on multivariate logistic regression analysis findings mentioned above, we firstly established a short-term recurrence risk nomogram model for glioma to provide individualized short-term recurrence prediction. Then, this model was validated by C-index, calibration, and ROC curves; and these verification results showed that our nomogram model had a good and reliable predictive function.

Several nomogram models for glioma were established to assess the survival probabilities in prior published studies, such as GBM, lower-grade glioma, and thalamic glioma nomogram models (13, 16–18). These models were not only applicable to American patients but were well validated in Asian populations (17), and we appreciated their contribution for helping us to gain better understanding of different types of gliomas. However, as we all know, time to recurrence had a significant influence on survival; therefore, it was important to predict the recurrence time of the glioma patients, especially for those patients at risk of short-term recurrence. Our recurrence risk model can help us estimate the short-term recurrence probability, and we can decide the interval of follow-up based on the predicted result. For example, for patients with a high short-term recurrence rate predicted by this model, we can shorten the follow-up time to detect whether the tumor has recurred and choose the appropriate treatment strategy according to the follow-up results. In addition, based on the predicted short-term recurrence probability, we can better communicate with the patient’s family, so that they have a better understanding of the patient’s status. And this model can give assistance to neurosurgeons and oncologists to assess the probability of short-term recurrence and formulate individualized treatment timely, which can lead to a better survival outcome for patients. In summary, we firstly established a short-term recurrence risk nomogram model based on a relatively large population to predict the short-term recurrence probability, and this model had a reliable predictive performance.

Limitations exist in our study. Firstly, all our patients received RT, and we could not analyze the influence of RT on tumor short-term recurrence; therefore, this nomogram may not be applicable to patients without RT after surgery. Secondly, MGMT promoter methylation status was a significant prognostic factor; however, its data were absent because most of the patients are not tested in our study, leading to the role of MGMT promoter status in our inability to assess short-term glioma recurrence. Thirdly, this risk model was only tested by internal validation; a larger sample is needed for external validation in the future study.

## Conclusion

A risk nomogram for estimating short-term recurrence risk for patients with glioma has been firstly developed. This tool offers an individualized prediction of short-term recurrence risk, and it provides an individual estimation instead of a group estimation. In order to facilitate the evaluation of short-term recurrence probability in clinical work, a free online nomogram model is provided (https://rj2021.shinyapps.io/Nomogram_recurrence-risk/). This tool can be used to decide the interval of follow-up time, offer disease counseling to patients, and give assistance to clinicians for developing an individualized management regimen.

## Data Availability Statement

The original contributions presented in the study are included in the article/**Supplementary Material**. Further inquiries can be directed to the corresponding author.

## Ethics Statement

The studies involving human participants were reviewed and approved by the ethics committees of Renji Hospital, School of Medicine, Shanghai Jiao Tong University.

## Author Contributions

Acquisition of data: TW, CZ, SZ, ZL, BC, KL, XY, and ZZ. Analysis and interpretation of data: TW, CZ, SZ, YB, YH, and ZW. Statistical analysis: TW, ZL, KL, and BC. Drafting the article: TW. Critically revising the article: TW, YM and RH. Funding acquisition: KL. Conception and design: TW and RH. Study supervision: RH. All authors contributed to the article and approved the submitted version.

## Funding

This work was supported by the National Natural Science Foundation of China (82002630).

## Conflict of Interest

The authors declare that the research was conducted in the absence of any commercial or financial relationships that could be construed as a potential conflict of interest.

## Publisher’s Note

All claims expressed in this article are solely those of the authors and do not necessarily represent those of their affiliated organizations, or those of the publisher, the editors and the reviewers. Any product that may be evaluated in this article, or claim that may be made by its manufacturer, is not guaranteed or endorsed by the publisher.
